# *M. tuberculosis *genotypic diversity and drug susceptibility pattern in HIV- infected and non-HIV-infected patients in northern Tanzania

**DOI:** 10.1186/1471-2180-7-51

**Published:** 2007-05-31

**Authors:** Gibson S Kibiki, Bert Mulder, Wil MV Dolmans, Jessica L de Beer, Martin Boeree, Noel Sam, Dick van Soolingen, Christophe Sola, Adri GM van der Zanden

**Affiliations:** 1 Department of Internal Medicine, Endoscopy Unit and Department of Medical Microbiology, Kilimanjaro Christian Medical Centre, Tumaini University, P.O Box 3010, Moshi, Tanzania; 2 Microbiology Laboratory Twente, Enschede, The Netherlands; 3 Department of Internal Medicine, Division General Internal Medicine, University Nijmegen Medical Centre St-Radboud, PO Box 9101 (484), 6500 HB Nijmegen, The Netherlands; 4 Medical Microbiology and Infection Control, Gelre Hospitals, Apeldoorn, The Netherlands; 5 Department of Pulmonary Diseases and University Lung Centre Dekkerswald, University Nijmegen Medical Centre St-Radboud, PO Box 9101 (484), 6500 HB Nijmegen, The Netherlands; 6 National Institute of Public Health and the Environment (RIVM), Bilthoven, The Netherlands; 7 Institut Pasteur de Guadeloupe, Guadeloupe; 8 Present address: Unité de Génétique Mycobactérienne, Institut Pasteur, Paris, France

## Abstract

**Background:**

Tuberculosis (TB) is a major health problem and HIV is the major cause of the increase in TB. Sub-Saharan Africa is endemic for both TB and HIV infection. Determination of the prevalence of *M. tuberculosis *strains and their drug susceptibility is important for TB control.

TB positive culture, BAL fluid or sputum samples from 130 patients were collected and genotyped. The spoligotypes were correlated with anti-tuberculous drug susceptibility in HIV-infected and non-HIV patients from Tanzania.

**Results:**

One-third of patients were TB/HIV co-infected. Forty-seven spoligotypes were identified.

Fourteen isolates (10.8%) had new and unique spoligotypes while 116 isolates (89.2%) belonged to 33 known spoligotypes. The major spoligotypes contained nine clusters: CAS1-Kili 30.0%, LAM11- ZWE 14.6%, ND 9.2%, EAI 6.2%, Beijing 5.4%, T-undefined 4.6%, CAS1-Delhi 3.8%, T1 3.8% and LAM9 3.8%. Twelve (10.8%) of the 111 phenotypically tested strains were resistant to anti-TB drugs. Eight (7.2%) were monoresistant strains: 7 to isoniazid (INH) and one to streptomycin. Four strains (3.5%) were resistant to multiple drugs: one (0.9%) was resistant to INH and streptomycin and the other three (2.7%) were MDR strains: one was resistant to INH, rifampicin and ethambutol and two were resistant to all four anti-TB drugs. Mutation in the *kat*G gene codon 315 and the *rpo*B hotspot region showed a low and high sensitivity, respectively, as predictor of phenotypic drug resistance.

**Conclusion:**

CAS1-Kili and LAM11-ZWE were the most common families. Strains of the Beijing family and CAS1-Kili were not or least often associated with resistance, respectively. HIV status was not associated with spoligotypes, resistance or previous TB treatment.

## Background

Tuberculosis (TB) persists as a major cause of morbidity and mortality, affecting almost a third of the world's population [1]. Inadequate detection and cure rates have been identified as reasons for a mounting global tuberculosis burden [2]. Human immunodeficiency virus (HIV) is by far the major cause of the current increase in tuberculosis infection. The presence of HIV increases the risk of reactivation of a latent *Mycobacterium tuberculosis *(MTB) infection [3] and rapid thus progression of the infection [4]; HIV also increases MTB transmission rates at the community level, therefore threatening the health and survival of HIV- seronegative individuals as well [5]. Sub-Saharan Africa is endemic for both TB and HIV infection [6], and pulmonary tuberculosis (PTB) in the HIV-affected countries of eastern and southern Africa, such as Tanzania, has increased rapidly in the past decades [7].

Molecular genotyping is an important tool for the understanding of TB epidemiology. It can predict transmission rate and identify dominant strains, strains with an enhanced capacity to spread, strains associated with outbreak [8], severe disease [9] and drug resistance. Spacer oligonucleotide typing (spoligotyping) is one of the molecular genotyping techniques; it is fast, robust, reliable, easy to perform, and cost-effective [10]. On the basis of the variability of the direct-repeat (DR) locus [10,11], spoligotyping can detect different *M. tuberculosis *strains, such as the Beijing genotype which is associated with enhanced febrile response in patients during treatment, and multiple drug resistance (MDR) [9,12,13].

For TB control, monitoring the emergence of drug resistant strains is essential. While detection of drug resistance by phenotype is hindered by the prolonged time to identify resistant strains, genotypic prediction of drug resistance is faster. Data are accumulating on the correlation between gene mutations and phenotypic resistance. Such data however are sparse, particularly from sub-Saharan Africa where the disease burden is highest. The primary mechanism for acquiring resistance in *M. tuberculosis *is the accumulation of point mutations in gene coding for drug targets or drug-converting enzymes [14]. Mutations in the catalase peroxidase gene (*kat*G) [15,16] and in a gene encoding the enoyl acyl carrier protein reductase (*inh*A) [17] have been found to account for 60 to 70% and 10 to 15% of INH-resistant MTB isolates, respectively [18]. Mutations resulting in an amino acid change within the 81-bp core region of the RNA polymerase β-subunit (*rpo*B) gene are found in 96% of rifampicin-resistant *M. tuberculosis *strains [19].

The objectives of this study were to determine the genetic biodiversity of MTB by spoligotyping and to correlate spoligotypes and anti-tuberculous drug susceptibility in HIV-infected and non-HIV patients in northern Tanzania, one of the TB and HIV endemic countries of eastern Africa.

## Results

### Characteristics of the study population

In total 130 positive-culture samples from 220 patients were included in the study. The majority of the patients in this study were from the Chagga and Masai tribes, 41.1% and 11.6%, respectively. Other tribes were represented by less than 5% each. Fifty-four (41.9%) were peasants, 28 (21.7%) business people, 18 (14%) miners and 10 (7.8%) herders, the remaining had other occupations. The mean age (SEM) was 37 years (1). Eighty-three (63.8%) were male patients. Fourteen patients (10.8%) had a history of previous treatment for tuberculosis. Forty-seven (36.2%) patients were HIV seropositive. Mean CD4 cell count (SEM) for the HIV seropositive patients was 200 (35) cells/ml and for the HIV seronegative group the mean CD4 count (SEM) was 555 (38) cells/ml.

### Spoligotypes

**Figure 1:**
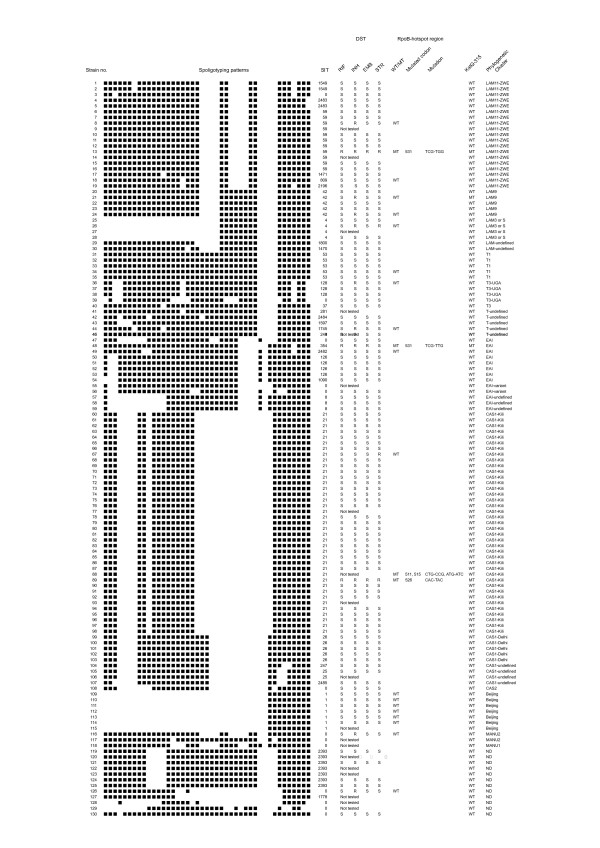
Description of *Mycobacterium tuberculosis *strains and clusters (SIT) found in Tanzania. DST = Drug Susceptibility Testing. SIT = spoligo-international type. WT = wild type. MT = mutant type.

A total of 47 spoligotypes was identified among the 130 *M. tuberculosis *isolates. Fourteen isolates (10.8%) had new and unique spoligotypes (SIT 0) while 116 isolates (89.2%) belonged to 33 previously known spoligotypes. A total of 106 isolates (81.5%) from nine different phylogenetic clusters formed the major isolates (i.e. with five or more isolates for each spoligotype). These were CAS1-Kili with 39 (30%) isolates (strains 60–98), LAM11- ZWE 19 (14.6%) (strains 0–19), ND 12 (9.2%)(strains 119–130), EAI 8 (6.2%)(strains 47–54), Beijing 7 (5.4%)(strains 109–115) and T-undefined 6 (4.6%)(strains 41–46) as well as CAS1-Delhi (strains 99–103), T1 (strains 31–35) and LAM9 (strains 20–24) with 5 isolates (3.8%) each (Figure 1).

Three new shared spoligotypes (a common pattern shared by two or more isolates) were established from our findings by comparing them with the spoligotypes which were available in the SpolDB4: one EAI (SIT 2482, strain 49) with that found in Zambia, one T-undefined (SIT 2482, or SIT 2484, strain 42) with that found in the USA and one CAS1 variant – undefined (SIT 2485, strain 107) with that found in Sweden.

### Drug susceptibility testing (DST)

Out of 111 TB isolates which were subjected to DST, 12 (10.8%) were resistant to at least one anti-TB drug. Eight of the 111 (7.2%) were monoresistant strains: 7 resistant to INH and one to streptomycin. Four strains (3.5%) were resistant to multiple drugs: one (0.9%) was resistant to INH and streptomycin and the other three (2.7%) strains were MDR strains: two (1.8%) strains were resistant to all four anti-TB drugs and one (0.9%) strain was resistant to INH, rifampicin and ethambutol Resistance of the 111 isolates for each drug was as follows: INH 11 strains (9.9%), streptomycin 4 (3.6%), rifampicin 3 (2.7%), and ethambutol 3 (2.7%).

Four of the 130 MTB isolates (3.1%) were found to have a mutation in a *kat*G gene codon 315. Four of the 22 tested isolates were found to have a mutation in the hotspot region of the *rpo*B gene. Of the 11 strains phenotypically resistant to INH, four strains were also genotypically resistant to INH. The sensitivity of the mutant *kat*G gene to predict phenotypical resistance to INH was 30.8% and the specificity was 100%.

All three strains phenotypically resistant to rifampicin were also genotypically resistant to rifampicin. Both sensitivity and specificity of a mutation in the *rpo*B gene to predict phenotypical resistance to rifampicin was 100%.

As shown in Table 1, two of the 49 isolates (4.1%) belonging to four variants of the CAS1 family (CAS1-Kili, CAS2, CAS1-undefined, CAS1-Delhi) showed resistance to at least one drug and four could not be tested. Six of the 30 isolates (20%) of the LAM family (LAM3 or S, LAM9, LAM11-ZWE) showed resistance to one or more anti-TB drugs. Three of the 16 isolates (18.8%) of the T family (T3-UGA, T1, T-undefined) showed resistance. One of the 13 isolates (7.7%) of the EAI family (EAI, EAI-variant, EAI-undefined), one of the 12 isolates (8.3%) of the ND family, and one of the three isolates (33.3%) from the MANU family (MANU1, MANU2) showed resistance to at least one anti-TB drug. None of the seven *M. tuberculosis *Beijing isolates was resistant to anti-TB drugs.

**Table 1:** Distribution of seven of the phylogenetic families by age, smear results, HIV status, CD4 count (n = 130) and phenotypic resistance for a TB drug (n = 111)

Family	Isolates		Age		Smear positive		HIV positive		CD4cell/ml		Resistant	
	N = 130		N = 130		N = 130		N = 130		N = 130		N = 111	
	%	n/N	mean	SEM	%	n/N	%	n/N	mean	SEM	%	n/N

CAS *	37.7	49/130	38	2	81.6	40/49	34.7	17/49	471	64	4.4	2/45
LAM **	23.2	30/130	39	3	83.3	25/30	26.7	8/30	437	55	18.5	5/27
T ***	12.3	16/130	35	2	81.3	13/16	50.0	8/16	436	92	14.3	2/14
EAI #	10.0	13/130	39	3	69.2	9/13	46.2	6/13	432	64	8.3	1/12
ND	9.2	12/130	37	4	58.3	7/12	41.7	5/12	413	99	16.7	1/6
Beijing	5.4	7/130	29	2	85.7	6/7	14.3	1/7	380	69	0.0	0/6
MANU ##	2.3	3/130	32	4	100	3/3	66.7	2/3	195	141	100	1/1

* CAS: CAS1-Kili; CAS1-Delhi; CAS1-undefined;CAS2

** LAM: LAM11-ZWE; LAM9; LAM3 or S; LAM-undefined

*** T: T-undefined; T1; T3-UGA; T3

# EAI: EAI; EAI-variant; EAI-undefined

## MANU: MANU1; MANU2.

Out of the 12 resistant isolates two (14.3%) were new and unique isolates (strain 116 and 126): the three MDR isolates were CAS1- Kili (strain 89), EAI (strain 48) and LAM 11 – ZWE (strain 13) (Figure 1)

### Previous TB treatment, HIV status and drug resistance

Out of 14 patients who had a history of previous treatment for PTB, one (7.1%) had a MDR-MTB strain and the other 13 (92.9%) had strains sensitive to all four first line anti-TB drugs. Five of the 47 HIV-infected individuals (10.6%) showed resistance to at least one drug. Three of the 47 HIV – infected individuals (6.4%) had a history of previous treatment for TB and none of them had strain resistant to anti-TB drug.

## Discussion

There was a wide diversity of spoligotypes in this study group; the 130 MTB isolates produced 47 different spoligotypes. The clades observed in this study were more than three-quarters of the total of 62 clades/lineages currently documented in the fourth international spoligotyping database, SpolDB4 [20]. This diversity may be attributed to increased human movement in this area due tourism, mining, asylum seeking (refugees) and transborder/international business. Historically, many people from Europe and Asia also lived here during pre- and post-independence times. The structure of the TB population is determined by geography, demography, and human migration. Studies have shown that the host's geographical origin is predictive of the clinical *M. tuberculosis *isolates and there is an apparent stable association of TB bacilli populations with their human hosts in various environments [21]. New types (i.e. orphan spoligotypes) accounted for one-tenth of all spoligotypes in this study. The low percentage of new types may also be a result of increased human movements. Countries with a history of isolation have been shown to have a large number of new spoligotypes [8]. In this study two of the three new shared spoligotypes were formed between two geographically widely separated isolates (strain 42 and 107). This again is more likely to be due to the increased human movement than homoplasy (i.e. acquisition of two similar structures without a common ancestor). If it is assumed that tuberculosis may have affected the early hominids in East Africa, evolved clonally and then spread to the rest of the world coincidentally with human migration out of Africa [13-16], then it is interesting to find that almost all strains go back to the origin of *Homo sapiens sapiens*. The site of this study is within the area of Olduvai Gorge in northern Tanzania, which is regarded as the "cradle of mankind". In this context, strains no. 116–118 with MANU-derived spoligotypes are of particular interest (Figure 1).

Among the different phylogenetic clusters found in this study, there were only nine major clusters with five or more isolates, but they comprised 81.5% of all isolates. This indicates the high transmission rate within the population. This high transmission rate may also be influenced by the marked prevalence of HIV infection which increases the risk of tuberculosis for all people in a community, both those with HIV infection and the HIV seronegative community members [5]. Two clusters were by far the most prevalent: CAS1-Kili and LAM11-ZWE family, with a prevalence exceeding ten percent. The predominance of these two families by more than ten percent has been demonstrated by another study based on IS *6110*-restriction fragment length polymorphism (RFLP). The study showed that the Kilimanjaro and Meru families were the most prevalent in this area [22], the names of the families being derived from the two adjacent mountains found in this area. While the Kilimanjaro family refers to CAS1-Kili, the Meru family is identical to LAM11-ZWE [20]. Another recent study, performed in Dar-es-Salaam, suggested the existence of new CAS1-Kili genotype variants (designated as CAS1-Dar) [23]. However, the absence of spacer 2 and 15 in the spoligotype-signatures reported by these authors was not found, even once, in our study. A single clinical isolate, strain 104 (ST247) looks similar to the CAS-Dar variants. This may be explained either by a specific loss of spacers 2 and 15 in the CAS1-Dar variants or by false negative hybridization spots. Spacer 2 and 15 may indeed sometimes provide barely interpretable results. If confirmed, the absence of spacers 2 and 15 may provide interesting clues about the microevolution of CAS1-Kili in Tanzania.

More than 5% of cases were Beijing isolates which were the fifth most frequent spoligotype. The presence of seven Beijing isolates had already been reported in Dar-es-Salaam [23]. The mean age of the patients with the Beijing family was the lowest compared to those with other isolates (Table 1). It has already been reported that Beijing is associated with young age [24] which implies recent and ongoing transmission.

A striking finding was that not one single case of *M. bovis *or *M. africanum *was found in this study. This study cohort consisted mainly of peasants and herders, including the Masai people who are essentially semi-nomads or herders and their staple food includes milk and meat. In Uganda which is a neighbouring country, other genotypes of the *M. tuberculosis *complex, previously designated as "*M. africanum 2" *are the major cause of human tuberculosis [25]. This finding may be a result of improved livestock and animal husbandry in Tanzania or due to anthropologically rooted differences between Uganda and Tanzania.

All resistant isolates except one were resistant to INH, either alone or in combination with other drugs. The level of INH resistance was high; a similar trend has been observed in many African countries; in some countries up to one-third of the isolates is resistant to INH [26-29]. This rate is alarming, especially since INH is the first-line drug which is used throughout the course of TB treatment. This also means a high probability of the development of MDR in the future, since it has been observed that MDR often develops from initial INH-monoresistant strains [28]. Several reports show a low prevalence of MDR-MTB in Africa compared to Asia and Eastern Europe [30]. But the trend seems to be steadily increasing since previous reports from Tanzania showed that INH monoresistance was about 5% and MDR was about 1% [31] while our study reveals a more than two-fold increase in both INH resistance and MDR. The upward trend in resistance has also been noted in neighboring countries [30,32].

Despite its high prevalence, the CAS 1 family showed the least association with anti-TB drug resistance (Table 1); family LAM and T were more frequently associated with anti-TB drug resistance.

Another interesting aspect was the fact that all MTB Beijing isolates in this study were susceptible to all anti-TB drugs unlike reports from other studies [28,33]. A previous study in Indonesia also showed no significant association between Beijing strains and drug resistance [9].

One-third of the study population was infected by both HIV and TB; this is the common trend in sub Saharan Africa [34]. The association of HIV infection, however, with previous treatment for TB was not statistically significant and that with anti-TB drug resistance was absent in this study. The smaller number of HIV- infected patients with a history of previous treatment for TB may be attributed to high mortality associated with the first MTB infection in TB/HIV co-infected patients [35,36]. There was no association between HIV status and spoligopatterns except for Beijing whereby only one person was HIV seropositive.

The mean CD4 count for patients with Beijing was the lowest, although all patients except one were HIV seronegative. This low CD4 count despite being HIV seronegative may be due to the pronounced virulence of the Beijing strain. It has been demonstrated that the Beijing family is more virulent than other *M. tuberculosis *strains. The virulence is attributed to the ability of the strain to produce phenolic glycolipid which is able to interfere with host immunity [37].

## Conclusion

In this study we found a wide diversity of spoligotypes with predominance of two genotype families. The Beijing family was also among the prevalent phylogenetic cluster. The most prevalent cluster CAS1-Kili and the most virulent Beijing were not or barely associated with anti-TB drug resistance.

A substantial proportion of resistant isolates was found, INH monoresistance being the highest and thus signaling the danger of increasing MDR in the future. MDR was higher than that described in previous reports from Tanzania.

## Methods

In the Kilimanjaro region of northern Tanzania, between April and September 2005, we collected sputum or bronchoalveolar lavage (BAL) fluid by direct expectoration or bronchoscopy, respectively, from 220 patients suspected of having PTB. The patients presented with coughing, evening fevers and abnormal chest x-rays. The patients were seen at Kibong'oto National Tuberculosis Referral Hospital (KNTH) and Kilimanjaro Christian Medical centre (KCMC), a tertiary care hospital. The specimens were obtained prior to initiation of TB treatment. For each patient, any history of TB treatment in the past was noted. Specimens were digested and decontaminated with NaLC and 2%NaOH by the standard procedure [38]. Sediments were used for diagnosis of TB: direct smears for acid-fast bacilli (AFB) by the Ziehl-Neelsen (ZN) method [38] and cultures in Löwenstein-Jensen (LJ) solid medium in three slants with or without 0.75% glycerol or with 0.6% sodium pyruvate. One hundred and thirty TB-positive culture samples (confirmed by smear) were subjected to spoligotyping and genotypic testing for drug resistance to isoniazid (INH) and 22 for rifampicin; 111 samples underwent phenotypic DST because the reculture of nineteen strains was not possible. Phenotypic drug resistance testing was performed for INH, rifampicin, ethambutol, and streptomycin and genotypic testing was carried out for a mutation in the *kat*G 315 codon to INH and for a mutation in the *rpo*B hotspot region to rifampicin. Genotypic resistance to rifampicin was tested with samples which were INH-resistant, contained phenotypic rifampicin-resistant strains, or had selected spoligotypes; phenotypic rifampicin -sensitive strains were used as controls.

### DNA extraction

A loopful of Mycobacterial colonies from cultures was suspended in 1 ml of physiological saline solution and heated at 80°C for 10 minutes to kill the bacilli. The supernatant obtained was stored at -20°C.

### Spoligotyping

Amplification of the spacers was performed using DNA extract and primers (DRa and DRb) corresponding to the direct repeat (DR) region of the genome of *M. tuberculosis *according to the procedure described by v.d. Zanden et al [39]. Briefly, 10 μl of the DNA extract were mixed with 40 μl PCR mix containing 5 μl 10× PCR buffer, 3 μl magnesium chloride (25 mM), 4 μl dNTP mix (2.5 mM dNTP each), 50 pmol DRa primer, 50 pmol DRb primer, 0.2 μl Taq polymerase and 0.25 μl Tris 1 M (pH 9.0), 0.5 μl UDG (1 U/μl). Amplification and hybridization were performed according to the manufacturer's manual (Isogen Bioscience BV, Maarssen, The Netherlands). The hybridized membrane was exposed to X-ray film for detection of the hybridization signal. The X-ray film (Hyperfilm™ ECL, Amersham Bioscience UK Ltd.) was read manually to obtain a complete pattern of the spacers between the DR regions harbored by a particular strain. Preventive measures were taken against contamination with either previously amplified DNA or amplicons from previous reactions; the procedure was described by Kwok *et al*. and Longo *et al; *respectively [40,41]. Computer-assisted analysis of spoligotyping patterns was carried out with Bionumerics 4.0 (Applied Maths, *St-Martin-Latem*, Belgium).

The patterns obtained received a spoligo-international type (SIT) according to the cluster assignment after the sequences were processed with the International Spoligotype Database of the Institut Pasteur de Guadeloupe [20,42]. New spoligotypes identified in the study were added to the SpolDB4.

### Phenotypic drug resistance testing

The proportional method for drug susceptibility testing (DST) of *M. tuberculosis*, as described in the manual, was used [43]; Briefly, for each drug a 1: 10 dilution of standardized inoculum was inoculated onto the control and drug-containing media. The extent of growth in the absence or presence of drug was compared and expressed as a percentage. If growth at the critical concentration of a drug was >1%, the isolate was considered to be clinically resistant. 7H10 agar with 0.2 and 1 mg/l isoniazid (INH), 1 en 5 mg/l rifampicin, 5 and 10 mg/l ethambutol and 5 and 10 mg/l streptomycin were used 7H10 agar tubes with 1 ml of culture in 9 ml sterile aquadest served as control.

### Genotypic INH and rifampicin resistance testing

Amplification of *kat*G gene containing codon 315 was performed for detection of INH resistance, using a set of primers; Foward (GGG CTT GGG CTG GAA GAG) and Reverse (ACA ACA GTT TCC TCG AGA TCC TGT). This resulted in a 122 bp long product. Detection of the *kat*G-315 wildtype took place by a FAM-labeled probe (5' CGCGATCACCAGCGGCATCG 3') and detection of the most common *kat*G-315 mutation from AGC to ACC by a VIC-labeled probe (5'CGC GAT CAC CAC CGG CAT CG 3'). DNA amplification/detection was performed in 30 μl reaction volume with the ABI Prism^® ^7000 Sequence Detection System (Applied Biosystems): template DNA, 400 nM of each primer and 200 nM of each probe. The PCR-program consisted of 2 min at 50°C, 10 min at 95°C, 45 cycles of 15 seconds at 95°C, 1 min at 60°C.

For the detection of rifampicin resistance, sequencing was carried out with the 3100-Avant sequencer (Applied Biosystems). Amplification of a 437 bp fragment, containing the *rpo*B-hotspot region, took place with a PTC 200 thermocycler (Biozym) by forward primer *rpo*B-F1 and reverse primer *rpo*B-R1, as described by v.d. Zanden et al. [19]. The sequence reaction was performed with approximately 50 ng DNA. The results of sequencing were compared to the *rpo*B-hotspot wildtype using Bionumerics software (Applied Maths, St-Martin-Latem, Belgium).

Blood was collected from all patients for detection of anti-HIV antibodies using the Capillus™ HIV-1/HIV-2 rapid test (Trinity Biotech, Bray, Ireland) according to the manufacturers instructions, with positive and negative controls for each test run. Samples positive in the rapid test were confirmed by ELISA using Vironostika^® ^HIV uniform II ag/ab microwell enzyme immunoassay (bioMerieux, Marcy 1'Etoile, France). CD4 counts were determined using flow-cytometry technique (Becton Dickinson Facs Count machine with BD Facscount™ reagent).

Data analysis was done by SPSS version 10-software for Windows. Normally distributed values were presented as mean plus standard deviation (SD). In other cases data were expressed as mean plus standard error mean (SEM). Chi-square was used to express correlations between dichotomous variables and Spearman's correlation coefficient, to quantify correlations between continuous variables. P-value equal to or less than 0.05 was regarded as statistically significant.

Ethical clearance was obtained from the Research Ethics Committee of KCMC hospital and a signed informed written consent was obtained from patients prior to enrolment of the patients in the study.

## Abbreviations

TB: tuberculosis

HIV: human immunodeficiency virus

MTB: *Mycobacterium tuberculosis*

PTB: pulmonary tuberculosis

DR: direct-repeat

MDR: multiple drug resistance

*Kat*G: catalase peroxidase gene

*Inh*A: enoyl acyl carrier protein reductase

*Rpo*B: RNA polymerase β-subunit

BAL: bronchoalveolar lavage

KNTH: Kibong'oto National Tuberculosis Referral Hospital

KCMC: Kilimanjaro Christian Medical Centre

AFB: acid-fast bacilli

ZN: Ziehl-Neelsen

LJ: Löwenstein-Jensen

INH: isoniazid

SIT: spoligo-international type

DST: drug susceptibility testing

PRIOR: Poverty Related Infection Oriented Research

WT: wild type

MT: mutant type
